# The cellular response of lipopolysaccharide-induced inflammation in keratoconus human corneal fibroblasts to RB-PDT: Insights into cytokines, chemokines and related signaling pathways

**DOI:** 10.1371/journal.pone.0318132

**Published:** 2025-01-27

**Authors:** Ning Chai, Tanja Stachon, Sabrina Häcker, Tim Berger, Zhen Li, Maryam Amini, Shweta Suiwal, Berthold Seitz, Achim Langenbucher, Nóra Szentmáry

**Affiliations:** 1 Dr. Rolf M. Schwiete Center for Limbal Stem Cell and Aniridia Research, Saarland University, Homburg/Saar, Germany; 2 Department of Plastic Surgery, The First Affiliated Hospital of Anhui Medical University, Hefei, China; 3 Department of Ophthalmology, Saarland University Medical Center, Homburg/Saar, Germany; 4 Experimental Ophthalmology, Saarland University, Homburg/Saar, Germany; 5 Department of Ophthalmology, Semmelweis University, Budapest, Hungary; Cedars-Sinai Medical Center, UNITED STATES OF AMERICA

## Abstract

**Purpose:**

Rose Bengal Photodynamic Therapy (RB-PDT) offers dual therapeutic benefits by enhancing corneal stiffness and providing antibacterial activity, presenting significant potential for patients with keratoconus complicated by keratitis. Our purpose was to assess the effect of rose bengal photodynamic therapy (RB-PDT) on the expression of pro-inflammatory cytokines and chemokines, as well as on extracellular matrix (ECM)-related molecules, in lipopolysaccharide (LPS)-induced inflammation of keratoconus human corneal fibroblasts (KC-HCFs). Additionally, the involvement of the mitogen-activated protein kinase (MAPK) and nuclear factor kappa B (NF-κB) signaling pathways which are downstream of the Toll-like receptor 4 (TLR4) pathway were examined.

**Methods:**

KC-HCFs were stimulated with varying concentrations of LPS (0–10 μg/ml), which was followed by RB-PDT. The expression levels of interleukin-1β (IL-1β), IL-6, IL-8, interferon alpha 2 (IFNA2), IFNB1, intercellular adhesion molecule 1 (ICAM-1), chemokine (C-C motif) ligand 4 (CCL-4), collagen I, collagen V, lysyl oxidase (LOX), transforming growth factor β 1(TGF-β1) were measured using qPCR, ELISA, or western blot. The activation of the NF-κB and MAPK pathways was assessed using qPCR and western blot.

**Results:**

In LPS-induced inflammation of KC-HCFs, the expression of IL-6 was further amplified by the treatment with RB-PDT (p = 0.001). However, the activation of the MAPK and NF-κB pathways did not increase following RB-PDT. Additionally, RB-PDT reduced the transcription of collagen I and collagen V (p≤0.03), while the transcription of LOX and TGF-β1 secretion remained unchanged in KC-HCFs exposed to LPS.

**Conclusion:**

In LPS-induced inflammation of KC-HCFs treated with RB-PDT, despite the increased expression of pro-inflammatory cytokines, the activation of the TLR4 signaling pathways is lacking. RB-PDT may have no adverse effects on corneal scar formation of keratoconus corneas in the short term.

## Introduction

Keratoconus is a bilateral ectatic disorder that progresses to thinning of the cornea and bulging of its central region, resulting in high/irregular astigmatism and impaired vision, often with corneal scarring [1,2]. The pathogenesis of keratoconus is multifactorial, encompassing environmental factors, genetic predisposition, age, and poor ocular hygiene habits [1]. Recent studies have also suggested a correlation between the development of keratoconus and the inflammatory status of the cornea, as evidenced by elevated levels of inflammatory cytokines such as interleukin-6 (IL-6) and tumor necrosis factor-α in patients’ tears [3,4]. Corneal fibroblasts are the primary cell type involved in the progression of keratoconus, as they are responsible for secreting extracellular matrix (ECM) and cytokines following stimuli such as corneal wounds or inflammation, thereby promoting corneal repair [5]. However, keratoconus human corneal fibroblasts (KC-HCFs) have exhibited distinct ECM secretory and regulatory patterns compared to healthy corneal fibroblasts, which may lead to an imbalance in the production and degradation of ECM in the corneal stroma [6].

In the context of therapeutic choices for keratoconus complicated by keratitis, clinicians may face a dilemma between the merits and drawbacks of conservative versus surgical treatments. During bacterial keratitis, proteases secreted by pathogens can directly damage the ECM [7]. Additionally, thinning of the corneal stroma and upregulation of matrix metalloproteinase levels in keratoconus patients may accelerate the progression of keratitis [8]. If the topical administration of antibiotics is ineffective, the risk of corneal melting and perforation increases. Conservative treatment of infectious keratitis does not address the progression of keratoconus. Corneal transplantation, conversely, as emergency surgical approach in keratitis poses a significantly greater risk of graft failure or the need for secondary optical keratoplasty than operating a quite eye [9]. Therefore, the considerations and decisions made by clinicians have significant implications for the disease outcomes of patients.

The treatments for keratoconus have been widely discussed in the literature. For patients with advanced keratoconus, penetrating keratoplasty is often necessary [10,11]. However, prior to this stage, photodynamic therapy (PDT) mediated corneal crosslinking has been discovered and subsequently employed in the treatment of keratoconus [12,13]. By topically applying a photosensitizer with specific wavelengths of light activation, corneal rigidity can be increased. In addition, PDT can also inhibit or eliminate various pathogens that cause keratitis, making it a potential treatment option for corneal infectious diseases [14–16].

Rose Bengal photodynamic therapy (RB-PDT) was initially reported by Cherfan *et al*. and has since been extensively investigated for its potential to increase corneal stiffness [17–19]. Furthermore, RB-PDT has demonstrated promising efficacy in keratitis caused by *Fusarium*, *Pseudomonas*, and *Staphylococcus aureus* [20–23]. Therefore, the dual therapeutic effects of RB-PDT in enhancing corneal stiffness and antibacterial activity hold significant potential for patients with keratoconus complicated by keratitis. Our previous study demonstrated that RB-PDT of lipopolysaccharide-stimulated human corneal fibroblasts (HCFs) can modify the inflammatory response by inducing interleukin-6 and interleukin-8 expression, and decreasing intercellular adhesion molecule-1 production [24]. The underlying mechanisms may be associated with nuclear factor kappa B and p38 mitogen-activated protein kinase pathway activation [24]. Nevertheless, the effects of RB-PDT on LPS-stimulated KC-HCFs have not been determined, yet. In keratoconus, chronic inflammation of the corneal tissue, inflammation related to dry eye disease or allergic conjunctivitis may all impact behavior of the cells [3,4]. In this study, we aimed to investigate the effects of RB-PDT on KC-HCFs complicated by keratitis, using an inflammatory model induced by lipopolysaccharide (LPS). Additionally, we examined the levels of proteins involved in nuclear factor kappa B (NF-κB) and mitogen-activated protein kinase (MAPK) pathways to explore the underlying molecular signaling mechanisms.

## Materials and methods

This study was granted approval by the Ethics Committee of Saarland, Germany (Approval No. 217/18), ensuring adherence to the ethical principles outlined in the Declaration of Helsinki. Prior to participation, all individuals provided written informed consent, reflecting their voluntary and informed decision to engage in this research. The keratoconus samples were collected between September 2019 and September 2021.

### 1. Cell isolation and cell culture

In the present study, five corneal samples from patients with keratoconus were collected from elective penetrating keratoplasties conducted at the Department of Ophthalmology, Saarland University Medical Center. The cell isolation process was conducted as previously described [25,26]. In brief, to isolate keratocytes, keratoconus corneal buttons were gently rinsed using phosphate-buffered saline (PBS, Merck, Sigma-Aldrich, Taufkirchen, Germany). Subsequently, these buttons were dissected into 5mm fragments using a surgical blade. The tissue fragments were then incubated in a solution comprising 1.0mg/ml collagenase A (Hoffmann-La Roche, Basel, Switzerland), along with Dulbecco’s modified Eagle’s medium (DMEM/F12, Thermo Fisher Scientific, Waltham, MA, USA). This medium was further supplemented with 5% fetal calf serum (FCS, Thermo Fisher Scientific, Waltham, MA, USA) and 1% penicillin-streptomycin (P/S, Sigma-Aldrich, St. Louis, USA) and incubated overnight at 37°C. Following incubation, the samples were centrifuged at 1500 rpm for 5min, and the supernatant was discarded. The resulting cell sediment was plated into a T75 flask containing 13ml of DMEM/F12 supplemented with 5% FCS and 1% P/S (referred to as the “basic medium” in the subsequent text). With the aid of FCS-containing culture medium, the keratocytes differentiated into corneal fibroblasts [27,28]. These cells were transferred to new T75 flasks for further experiments when they achieve approximately 80% confluence. The cell passages 2 to 5 were used for the experiments.

### 2. LPS treatment and RB-PDT

In this study, LPS derived from *Escherichia coli* was used (O26:B6, Cat No: L2654, Sigma-Aldrich, St. Louis, MO). The KC-HCFs were seeded onto either 96-well plates (designated for the XTT assay) or T75 flasks (for subsequent qPCR, ELISA, and Western blot analyses). When KC-HCFs reached approximately 60% confluence in the basic medium, the existing medium was discarded and various concentrations of LPS ranging from 0 to 10 μg/mL, were prepared in fresh basic medium and administered to each well (flask). The cells were then incubated for 24 hours to expose KC-HCFs to LPS.

To prepare RB solution, RB stock powder (C.I. 45440, Carl Roth, Karlsruhe, Germany) was used and reconstituted in basic medium with a concentration of 0.001 (m/v). After sterilization using a 0.2 μm filter, the obtained RB solution was aliquoted and stored in a 4°C refrigerator with lightproof condition for up to one month.

The illumination device was kindly provided and calibrated by the Experimental Ophthalmology Department of Saarland University in Homburg/Saar, Germany, for the light activation process. To perform RB-PDT, the LPS-containing basic medium was discarded, and the cells were gently rinsed with PBS to remove residual LPS. Subsequently, a 0% RB solution (for the control group or LPS-only group) and a 0.001% RB solution (for the RB-PDT only group and RB-PDT + LPS group) were added to wells or flasks for a 30-minute incubation at 37°C, followed by rinsing with PBS twice. Then, PBS was added again to KC-HCFs, and the cell culture was placed into the illumination device for 8 minutes of green light exposure (565 nm, 0.283 mW/cm^2^) to achieve a fluence of 0.14 J/cm^2^. Based on our preliminary experiments, this fluence intensity, following 0.001% RB exposure of the cells can prevent KC-HCFs from undergoing cell death after exposure to LPS and RB-PDT treatment. The PBS was then removed, and the basic medium was refilled for another 24-hour incubation at 37°C. The experimental groups measured in this study are described in detail below.

In brief, in the control group, KC-HCFs were cultured in basic medium. In contrast, the other groups used medium containing LPS. The cells in the control group underwent medium refreshment with basic medium devoid of LPS. During the RB-PDT treatment, the control group was similarly rinsed twice with PBS and incubated at 37°C for 10 minutes in PBS. Subsequently, the culture medium was replaced with basic medium.

In the RB-PDT only group, KC-HCFs were initially cultured in basic medium. While the other groups employed medium containing LPS, the RB-PDT only group underwent a medium refreshment with basic medium lacking LPS. During the RB-PDT treatment, the RB-PDT only group was rinsed twice with PBS. Subsequently, PBS was added again, and the cells were exposed to green light using the illumination device. Afterward, the culture medium was replaced with basic medium.

In the LPS only group, KC-HCFs were cultured in basic medium. When 60% confluence was reached, basic medium containing different concentrations of LPS was used for 24 hours of incubation. During the RB-PDT treatment, the LPS only group was rinsed twice with PBS. Subsequently, PBS was added again, and the cells were incubated at 37°C for 10 minutes. Afterward, the culture medium was replaced with basic medium again.

In the LPS + RB-PDT group, KC-HCFs were initially cultured in basic medium. When 60% confluence was reached, basic medium containing different concentrations of LPS was applied for 24 hours. During the RB-PDT treatment, the LPS + RB-PDT group was rinsed twice with PBS. Subsequently, PBS was added again, and the cells were exposed to green light using the illumination device. Afterward, the culture medium was replaced with basic medium.

### 3. Viability assay

Cell viability was assessed using the XTT assay (No. 11465015001, Roche, Sigma-Aldrich, Mannheim, Germany) 24 hours after treatment. Prior to use, the XTT labeling solution and the electron coupling reagent were freshly prepared and promptly mixed, with 50 μL of the mixed solution dispensed into each well. The cell plates were then incubated at 37°C for 3 hours. Following incubation, the optical density (OD) of each well was quantified using a Tecan Infinite F50 Absorbance Microplate Reader (TECAN Deutschland GmbH, Crailsheim, Germany) at a wavelength of 450 nm, with a reference wavelength of 690 nm.

### 4. Quantitative PCR

KC-HCFs were collected 24 hours after treatment in all groups, and the RNA was extracted using the Total RNA Purification Plus Micro Kit (cat. no. 48500, Norgen Biotek, Thorold, Canada). The genomic DNA (gDNA) was removed during the extraction, and the concentration of total RNA was measured using a Scandrop spectrophotometer (Analytik Jena AG, Jena, Germany).

Subsequently, reverse transcription was conducted using the One Taq^®^ RT-PCR Kit (E5310S, New England Biolabs Inc., Frankfurt, Germany). The obtained cDNA was stored at -20°C for subsequent measurements and analyses.

Quantitative PCR (qPCR) analysis was performed using the QuantStudio 5 Real-Time PCR System (ThermoFisher Scientific™ GmbH, Dreieich, Germany). Primers used for the qPCR analysis are detailed in **Table 1**. Each qPCR reaction was conducted in duplicate, employing the AceQ SYBR qPCR Master Mix (Vazyme Biotech, Nanjing, China) and 1 μL of cDNA template. To determine the relative expression of the target genes, the 2^−ΔΔCt^ method was adopted. For normalization purposes, the TATA-binding protein (TBP) and glucuronidase beta pseudogene (GUSB) were selected as endogenous controls, and the mean Ct values of these two reference genes were utilized accordingly.

**Table 1:** Primers used for qPCR.

Targeted cDNA	Gene symbol	Qiagen Cat. No.
IL-1β	IL1B	QT00021385
IL-6	IL6	QT00083720
IFN α2	IFNA2	QT00212527
IFN β1	IFNB1	QT00203763
IL-8	IL8	QT00000322
ICAM-1	ICAM1	QT00074900
CCL-4	CCL4	QT01008070
Collagen I	COL1A1	QT00037793
Collagen V	COL5A1	QT00044527
Lysyl oxidase	LOX	QT00017311
TGF-β1	TGFB1	QT00000728
IκB kinase subunit beta	IKBKB	QT00062482
NF-κB p65	RELA	QT02324308
ERK 1	MAPK3	QT02589321
ERK 2	MAPK1	QT00065933
p38	MAPK14	QT00079345
JNK	MAPK8	QT00091056
TATA box binding protein	TBP	QT00000721
GUSB	GUSB1	QT00046046

### 5. Enzyme-linked immunosorbent assays (ELISAs)

The cytokine concentration in the supernatant of KC-HCFs was measured using ELISA. In brief, twenty-four hours after treatment, the cell supernatant was collected from all groups, and was immediately stored at -80°C. The DuoSet^®^ ELISA kit from R&D Systems (Minneapolis, MN, USA) was employed for quantifying the concentrations of Interleukin-6 (IL-6, DY206-05), interleukin-8 (IL-8, DY208-05), chemokine (C-C motif) ligand 4 (CCL-4, DY271-05) and transforming growth factor beta 1 (TGF-β1, DY240-05) in the cell culture supernatant.

The measurement was conducted according to manufacturer’s protocol. Initially, the capture antibody was coated on each well of the plate overnight at room temperature. Subsequently, 100 μL of the collected supernatant was added to each respective well and was incubated for 2 hours. Following this, the detection antibody was introduced and incubated for a further 2 hours to enable the formation of an antibody-analyte-detection antibody sandwich complex.

For quantitative assessment, human recombinant proteins were used as standards to establish a calibration curve. The OD value of each well was measured using the Tecan Infinite F50 Absorbance Microplate Reader. The OD values were then normalized by dividing them with the total protein concentration of the respective flask, expressed as picogram (pg) per milligram of protein (mg). These normalized values were subsequently utilized for further statistical analysis.

### 6. Western blot

After the complete treatment series, the cell pellet was collected and lysed in RIPA buffer (R0278, Sigma-Aldrich, St. Louis, MO, USA) containing phosphatase inhibitors (cat. no. ab201112, Abcam, Cambridge, UK) and protease inhibitors (cat. no. 78430, Thermo Fisher Scientific, Waltham, MA, USA) to preserve protein integrity. The protein concentration in the lysates was determined using the Pierce™ BCA Protein Assay Kit (cat. no. 23227, Thermo Fisher Scientific, Waltham, MA, USA). The samples, containing 20 mg of total protein each, were then prepared for electrophoresis by boiling in loading buffer (cat. no. #1610747, Bio-Rad Laboratories, Hercules, CA, USA).

The proteins were separated on a 4–12% NuPage™ Bis-Tris SDS Gel (NP0321BOX, Invitrogen, Waltham, MA) and transferred onto 0.2 μm nitrocellulose membranes (cat. no. #1704158, Bio-Rad Laboratories, Hercules, CA, USA) using the Trans-Blot Turbo Transfer System (Bio-Rad Laboratories, Hercules, CA, USA). To normalize the protein loading, membranes were incubated in No-Stain™ Protein labeling reagent (cat. no. A44717; Waltham, MA, USA) for total protein normalization (TPN) and imaged using the iBright™ FL1500 Imaging System (Invitrogen, Waltham, MA, USA).

The membranes were then probed with specific antibodies, as listed in **Table 2**, to detect the proteins of interest. The immuno-reactive bands were visualized using Western Lightning Plus Chemiluminescence Reagent (NEL103001EA, PerkinElmer Inc., Waltham, MA, USA), and the signals were captured using the iBright™ FL1500 Imaging System. The band intensities were analyzed using the iBright™ Analysis Software 5.0 and normalized to the total protein amount, as visualized by No-Stain™ Protein labeling, to ensure accurate quantitative comparisons between samples. The resulting data were used for further analysis.

**Table 2:** Antibodies used for Western blot.

Antibody	Cat. No.	Manufacturer	Dilution	Incubation Time (4°C)
ICAM-1	#4915	Cell Signaling Technology	1:1000	Overnight
COL1A1	E6A8E	Cell Signaling Technology	1:1000	Overnight
COL5A1	ab7046	Abcam	1:1000	Overnight
LOX	D8F2K	Cell Signaling Technology	1:1000	Overnight
p-NF-κB p65	#93H1	Cell Signaling Technology	1:1000	Overnight
NF-κB p65	#8242	Cell Signaling Technology	1:1000	Overnight
p-ERK	#4370	Cell Signaling Technology	1:1000	Overnight
ERK	#4695	Cell Signaling Technology	1:1000	Overnight
p-p38	#4511	Cell Signaling Technology	1:1000	Overnight
p38	#8690	Cell Signaling Technology	1:1000	Overnight
p-JNK	#4668	Cell Signaling Technology	1:1000	Overnight
JNK	#9252	Cell Signaling Technology	1:1000	Overnight

### 7. Statistical analysis

Statistical analyses were conducted using GraphPad Prism 9.2 software (GraphPad Software, San Diego, CA). For comparing groups, a two-way ANOVA followed by Fisher’s exact test has been employed. The data were subsequently presented as the mean ± standard error of the mean (SEM), and a p-value less than 0.05 was considered statistically significant.

## Results

### 1. LPS stimulation combined with RB-PDT impaired KC-HCFs viability

**Fig 1:**
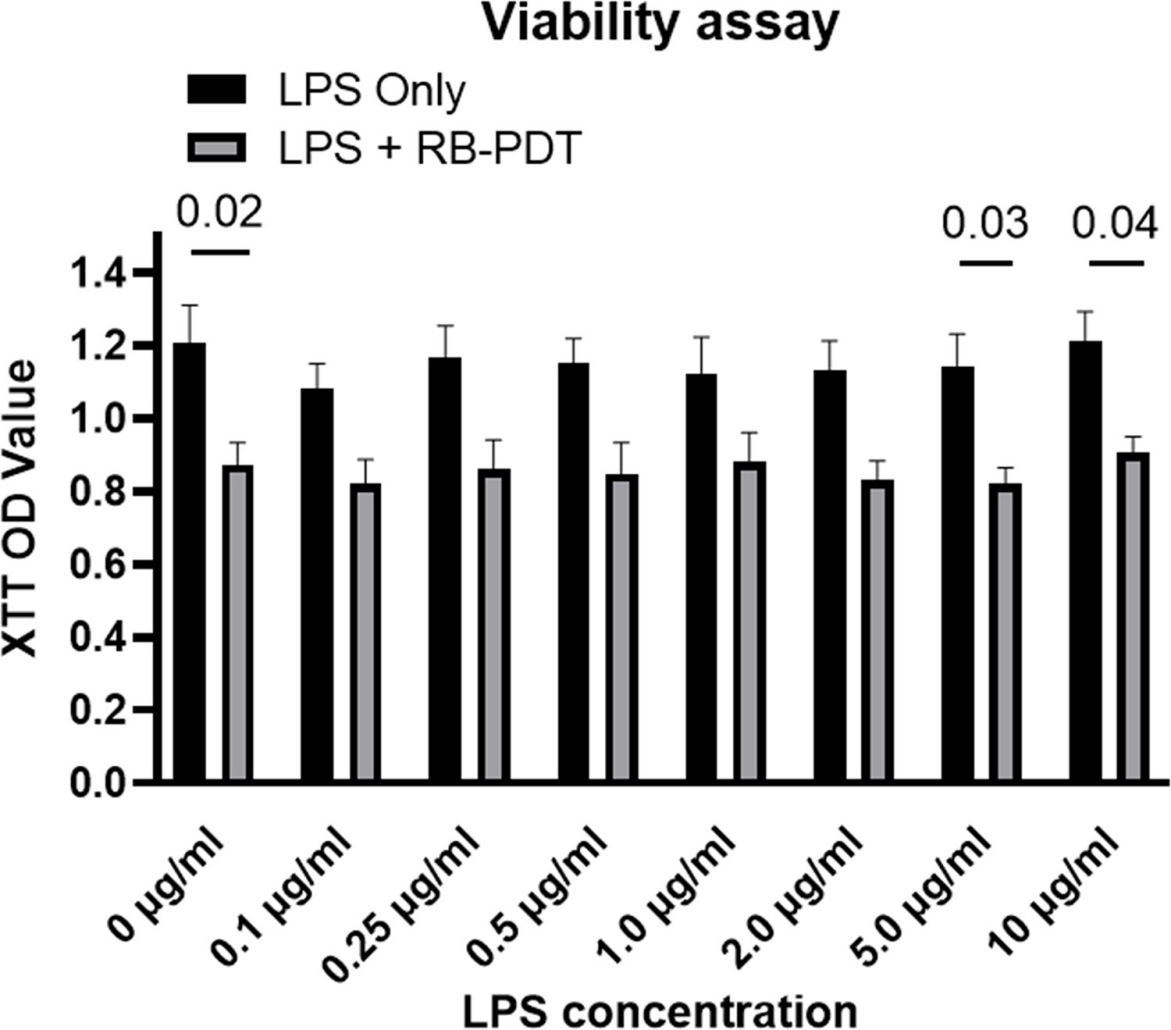
Keratoconus human corneal fibroblast (KC-HCFs) viability 24 hours after either 0–10 μg/ml lipopolysaccharide (LPS) treatment only or in combination with subsequent rose-bengal photodynamic therapy (RB-PDT) (n = 5). Measurements have been performed in duplicate. Data are shown as mean ± standard error of the mean. Two-way ANOVA has been performed, and p values below 0.05 were considered statistically significant. KC-HCFs viability was significantly lower using 0, 5.0, 10 μg/ml LPS combined with RB-PDT, than using LPS only with the same concentration (p≤0.04).

The viability of KC-HCFs was assessed 24 hours after this series of treatments, as depicted in **Fig 1**. RB-PDT alone reduced cell viability compared to the control group (p = 0.02). When LPS was administered at concentrations ranging from 0.1 to 2.0 μg/mL, no significant difference in cell viability was observed between the LPS only group and the group treated with LPS combined with RB-PDT (p≥0.05). However, as the LPS concentration increased to 5.0 μg/mL, the combination of LPS and RB-PDT significantly impaired cell viability compared to the LPS-only group. To ensure comparability of the LPS only group and the LPS+RB-PDT group, without changing cell viability, we have consequently selected 0.1 μg/mL and 2.0 μg/mL LPS concentration as experimental conditions for subsequent measurements.

### 2. Effect of RB-PDT on pro-inflammatory cytokine expression in LPS stimulated KC-HCFs

**Fig 2:**
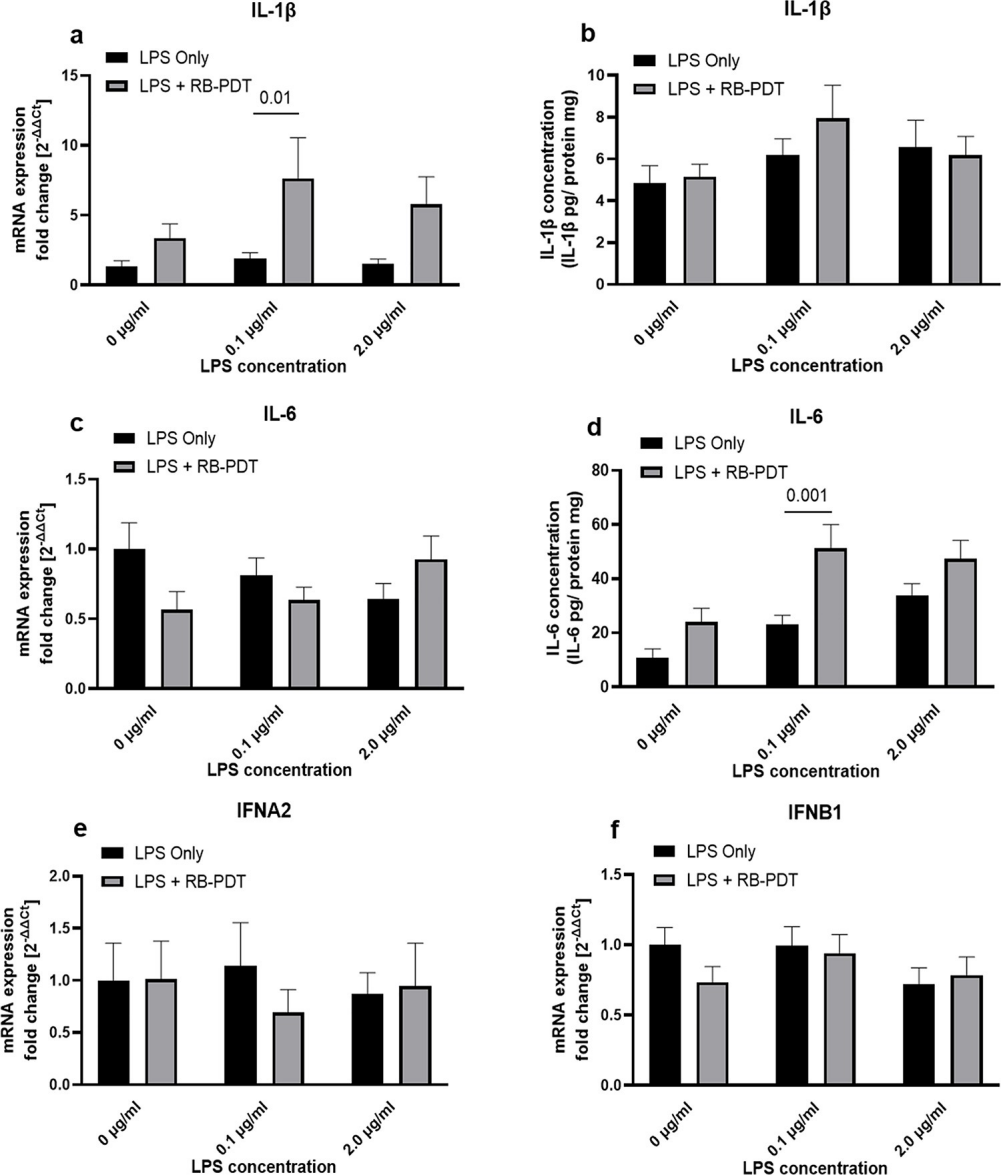
Rose-bengal photodynamic therapy (RB-PDT) promotes the expression of interleukin (IL) -1β and IL-6 in LPS induced KC-HCFs inflammation (n = 5) (a-f). Measurements have been performed in duplicate. Data are shown as mean ± standard error of the mean. Two-way ANOVA has been performed, and p values below 0.05 were considered statistically significant. Twenty-four hours after RB-PDT, IL-1β transcription was upregulated (**a**, p = 0.01), and the IL-6 protein concentration in the KC-HCFs supernatant was increased (**d**, p = 0.001). Nevertheless, IFNA2 and IFNB1 mRNA expression did not differ significantly between any of the groups (p≥0.14).

24 hours after RB-PDT, the expression of pro-inflammatory molecules, including IL-1β, IL-6, interferon alpha 2 (IFNA2), and IFNB1, were assessed (**Fig 2A–2F)**. Upon stimulation of KC-HCFs with a combination of 0.1 μg/mL LPS and RB-PDT, a significant increase in IL-1β transcription was observed compared to the LPS only group (**Fig 2A**, p = 0.01). Furthermore, IL-6 concentration in the culture supernatant of KC-HCFs, exposed to 0.1 μg/ml LPS and treated by RB-PDT was significantly elevated, compared to the LPS only group (**Fig 2D**, p = 0.001). However, RB-PDT did not exhibit significant effect on the transcription level of IFNA2 and IFNB1.

### 3. Effect of RB-PDT on chemotactic cytokine expression in LPS induced KC-HCFs

**Fig 3:**
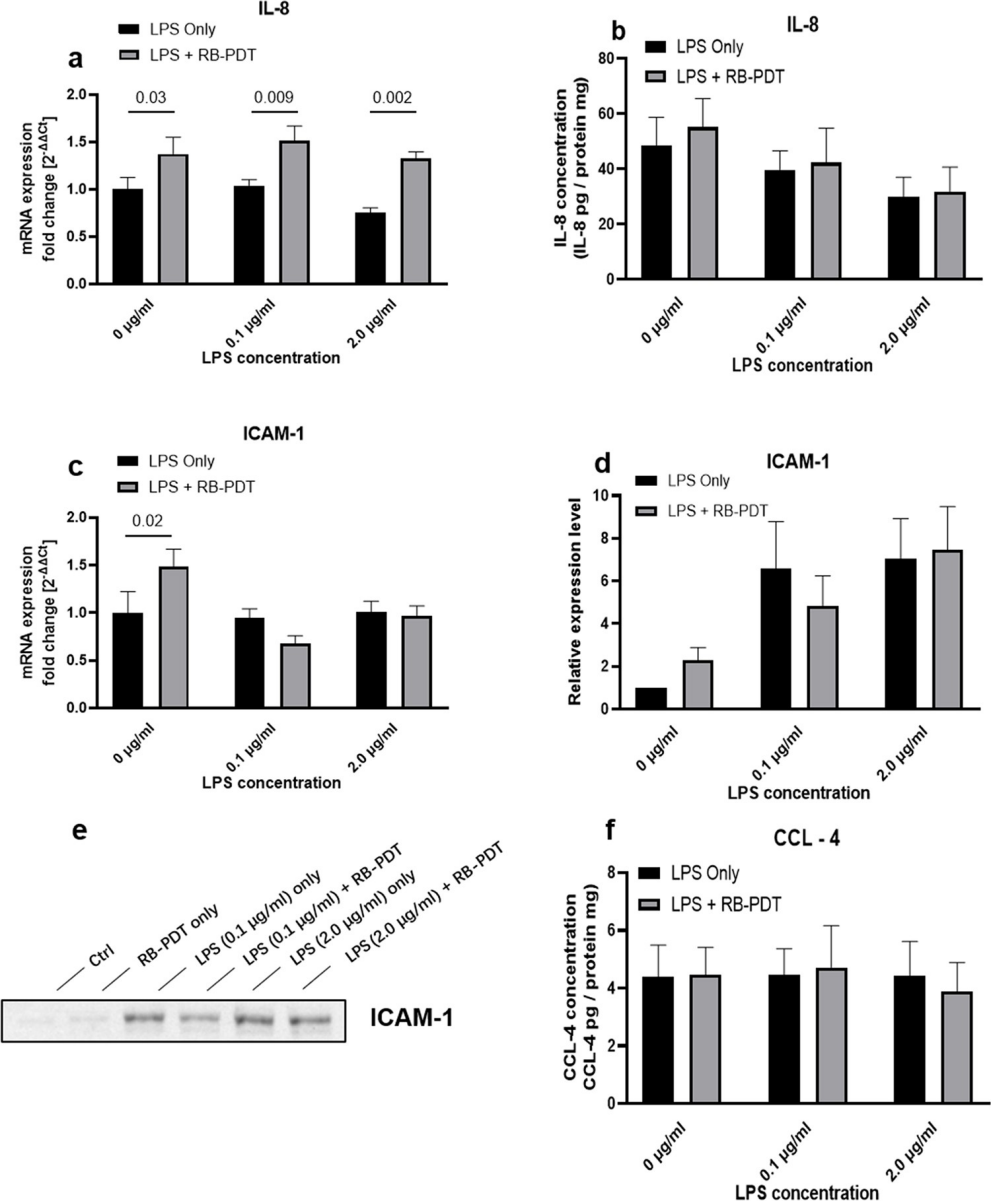
Interleukin (IL)-8, intercellular adhesion molecule (ICAM)-1 and chemokine (C-C motif) ligand (CCL)-4 expression in LPS-induced KC-HCFs inflammation, with subsequent rose-bengal photodynamic therapy (RB-PDT) (n = 5) (a-f). Measurements have been performed in duplicate. Data are shown as mean ± standard error of the mean. Two-way ANOVA has been performed, and p values below 0.05 were considered statistically significant. Twenty-four hours after RB-PDT, IL-8 transcription significantly increased in KC-HCFs exposed to 0–2.0 μg/ml LPS (**a**, p≤0.03). Additionally, RB-PDT upregulated ICAM-1 transcription in KC-HCFs without LPS stimulation (**c**, p = 0.02). However, RB-PDT did not affect IL-8 and CCL-4 concentrations in the supernatant of LPS-treated KC-HCFs (**a, f**), or modified ICAM-1 protein expression levels (**d, e**).

The effect of RB-PDT on expression of chemokines in LPS-exposed KC-HCFs is presented at **Fig 3**. Compared to the control group, the transcription level of IL-8 significantly increased following RB-PDT (**Fig 3A**, p = 0.03). Similar increase was observed in KC-HCFs exposed to 0.1 and 2.0 μg/mL LPS, followed by RB-PDT (**Fig 3A**, p≤0.002), indicating that RB-PDT upregulated the transcriptional capacity of IL-8 in the LPS-induced KC-HCFs. Furthermore, RB-PDT also promoted the transcription of another chemokine, ICAM-1 (**Fig 3C**, p = 0.02), in KC-HCFs without LPS exposure. Notably, at protein level, RB-PDT did not demonstrate a modulating effect on the expression of IL-8, ICAM-1, or CCL-4 in the 0.1 and 2.0 μg/mL LPS-induced KC-HCFs.

### 4. RB-PDT modulated the expression of extracellular matrix related molecules in LPS-stimulated KC-HCFs

**Fig 4:**
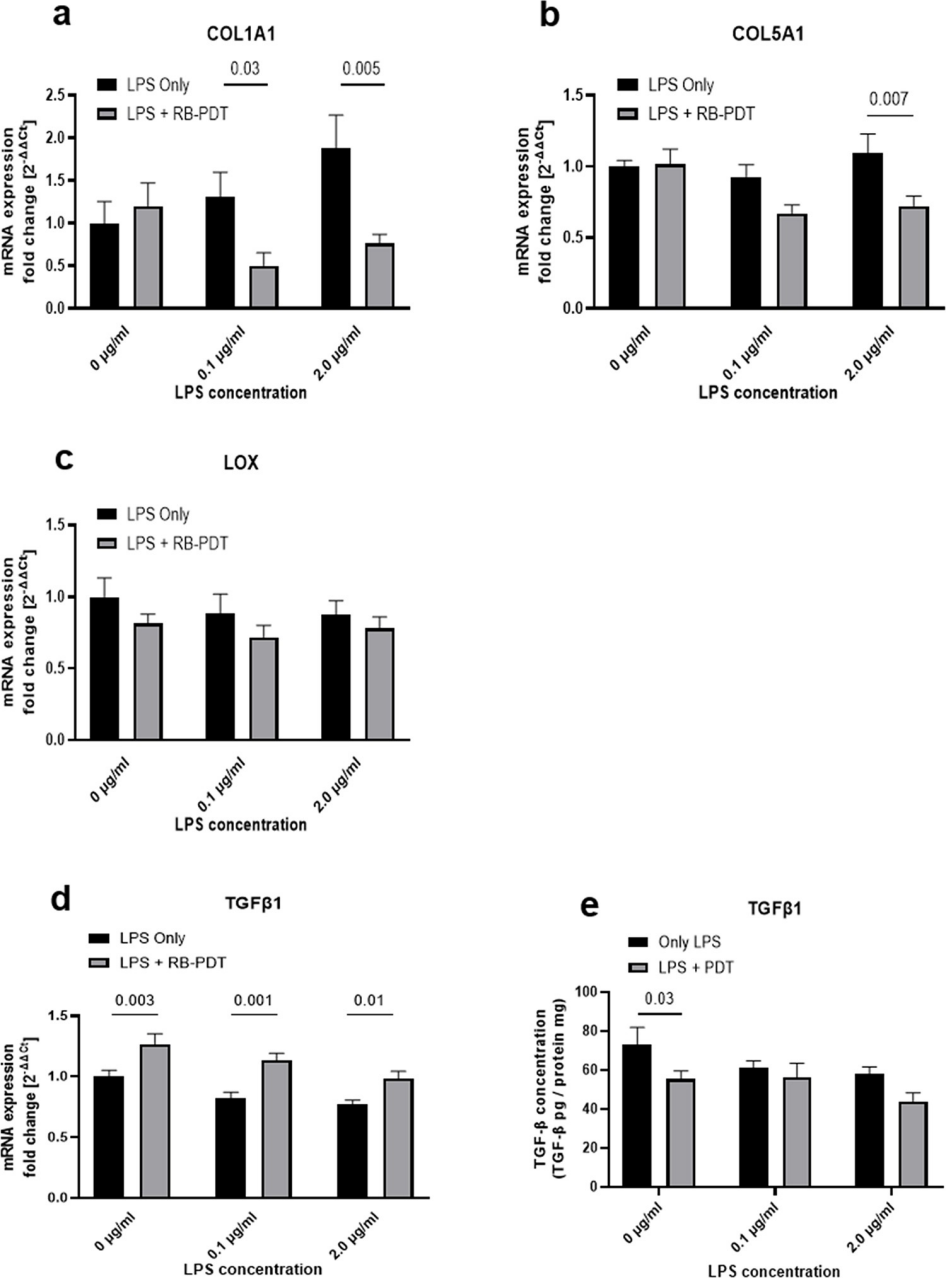
The effect of rose-bengal photodynamic therapy (RB-PDT) on the extracellular matrix-related molecules collagen I and V, lysyl oxidase (LOX) and transforming growth factor Beta 1 (TGF-β1) in LPS-exposed KC-HCFs (n = 5) (a-d). Measurements have been performed in duplicate. Data are shown as mean ± standard error of the mean. Two-way ANOVA has been performed, and p values below 0.05 were considered statistically significant. RB-PDT downregulated Collagen I and Collagen V transcription in 0.1 and 2.0 μg/ml LPS-treated KC-HCFs (**a, b**; p≤0.03), while lysyl oxidase (LOX) mRNA expression remained unaffected (**c,** p≥0.21). TGF-β1 transcription increased significantly in RB-PDT treated KC-HCFs, without and with LPS stimulation (**d**, p≤0.01). TGF-β1 concentration in the cell culture supernatant was significantly lower after RB-PDT, without LPS simulation (**e**, p = 0.03).

KC-HCFs are responsible for secreting numerous ECM related molecules, including collagen I, collagen V, and lysyl oxidase (LOX). TGF-β1 can affect and modulate the synthesis of ECM in the corneal stroma, and exerts a stimulatory effect on cell differentiation. The expression level of these molecules 24 hours after LPS treatment in combination with RB-PDT is demonstrated at **Fig 4**. The results indicated a significant downregulation in the transcription of collagen I in KC-HCFs exposed to LPS and subsequently treated by RB-PDT (**Fig 4A,** p≤0.03). Similarly, the transcription of collagen V was also suppressed by RB-PDT in KC-HCFs stimulated with 2.0 μg/mL LPS (**Fig 4B,** p = 0.007). However, the transcription level of LOX remained unchanged following RB-PDT treatment (p≥0.21). We further conducted Western blot analyses to quantify the protein expression level of collagen I, collagen V, and LOX. Nonetheless, these protein bands were not detectable in the analyzed groups, only at positive controls. In presence or absence of LPS, the transcription of TGF-β1 in KC-HCFs was enhanced by RB-PDT (**Fig 4D**, p≤0.01). Furthermore, TGF-β1 concentration in the cell culture supernatant was significantly lower after RB-PDT, without LPS simulation (**Fig 4E,** p = 0.03).

### 5. RB-PDT does not affect NF-κB and MAPK signaling pathways in LPS-induced KC-HCFs

**Fig 5:**
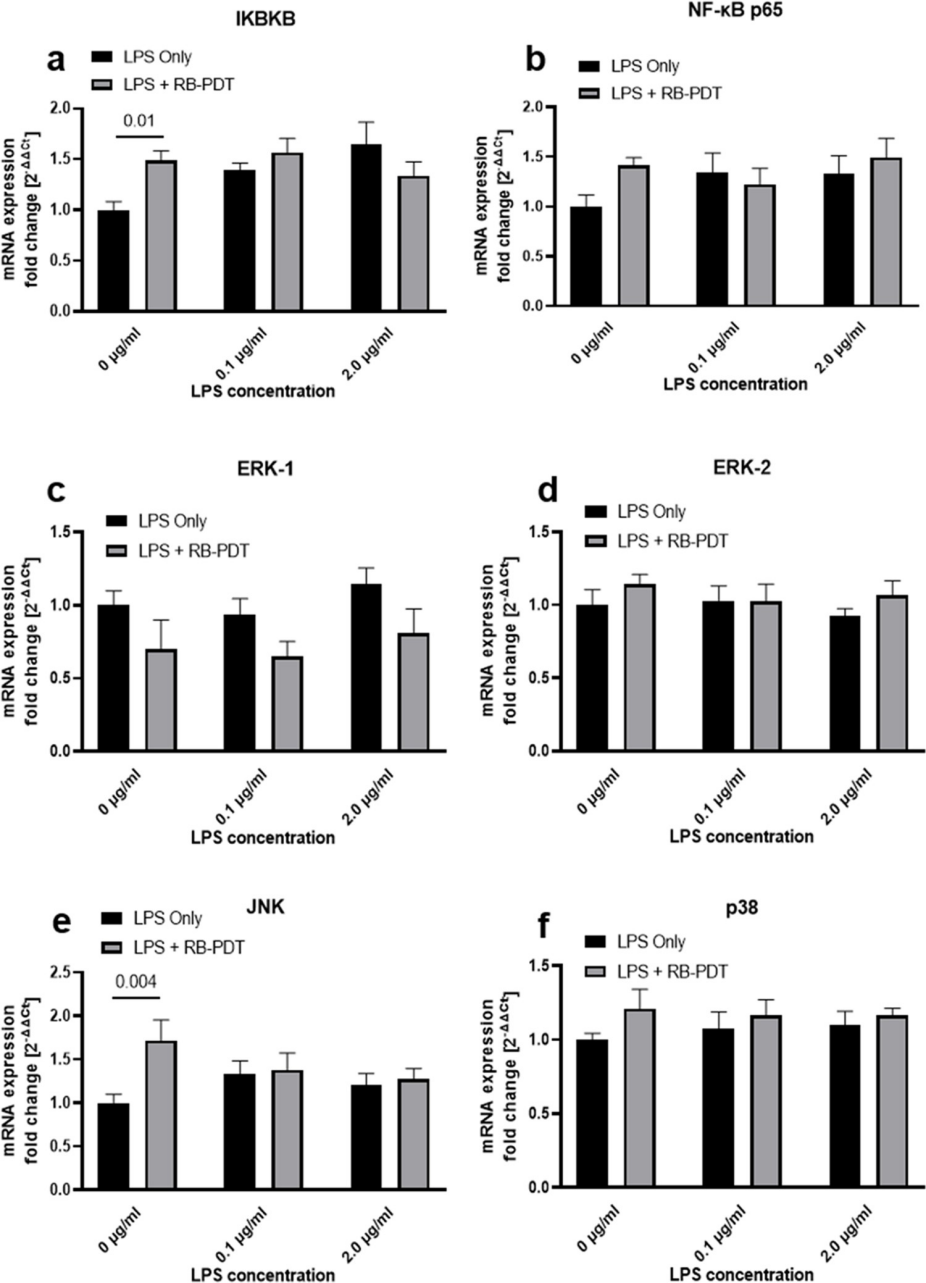
The transcription of nuclear factor kappa B (NF-κB) and mitogen-activated protein kinase (MAPK) signaling pathways after LPS treatment in combination with rose-bengal photodynamic therapy (RB-PDT) (n = 5) (a-f). Measurements have been performed in duplicate. Data are shown as mean ± standard error of the mean. Two-way ANOVA has been performed, and p values below 0.05 were considered statistically significant. Twenty-four hours after RB-PDT, the transcription of IκB kinase subunit beta (IKBKB) and c-Jun amino terminal kinase (JNK) was upregulated in KC-HCFs without previous LPS exposure (**a, e;** p≤0.01). However, RB-PDT without or with LPS exposure did not affect the transcription of IKBKB, NF-κB p65, extracellular signal-regulated kinases (ERK)-1, ERK-2, JNK, and MAP kinase p38 (p38) in KC-HCFs (**a-f,** p≥0.09).

**Fig 6:**
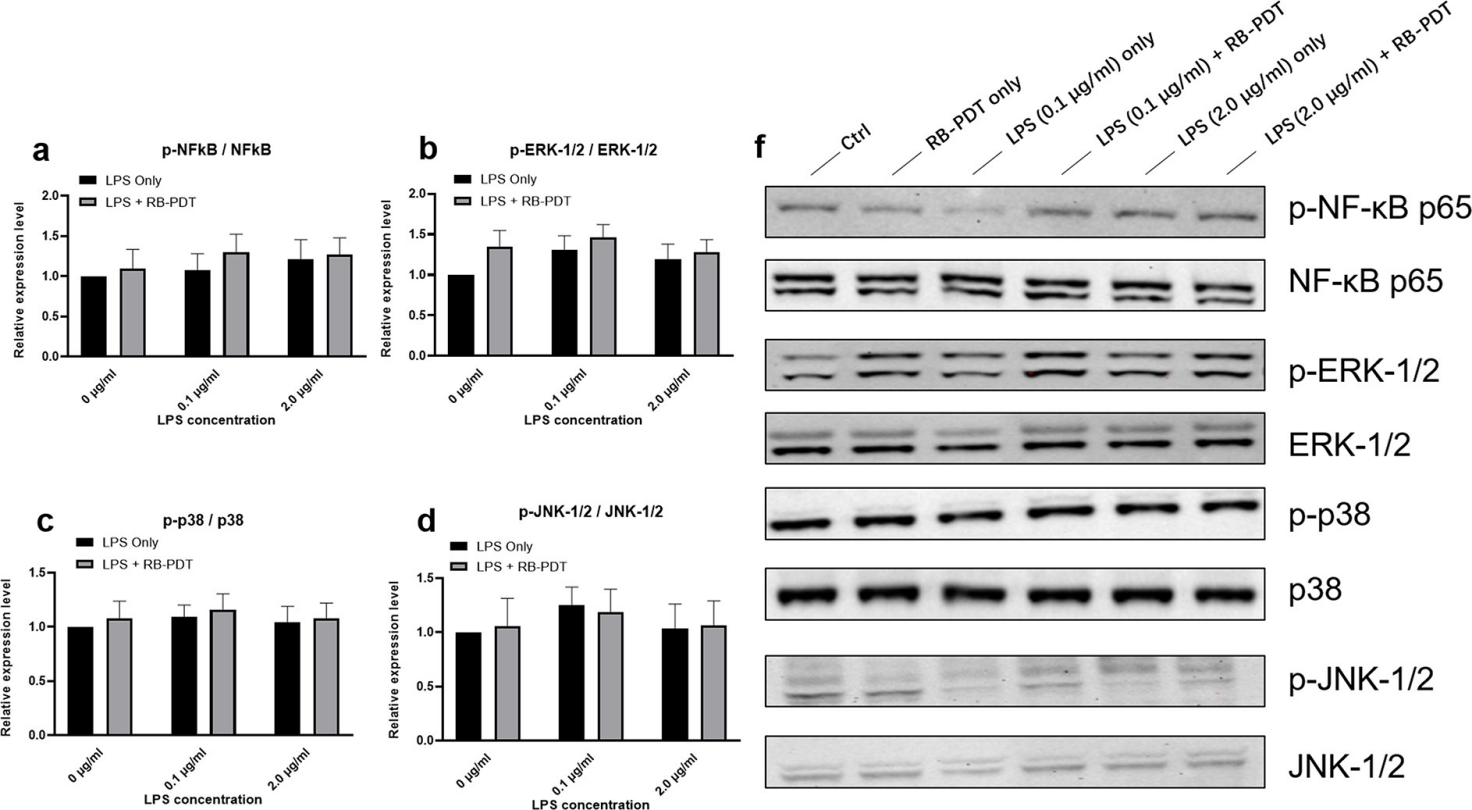
Activation of NF-κB and MAPK signaling pathways after LPS treatment in combination with subsequent rose-bengal photodynamic therapy (RB-PDT) (n = 5) (a-f). Measurements have been performed in duplicate. Data are calculated according to the ratio of phosphorylated protein and total protein and shown as mean ± standard error of the mean. Two-way ANOVA has been performed, and p values below 0.05 were considered statistically significant. NF-κB, ERK1/2, p38, and JNK 1/2 activation was not affected by RB-PDT in KC-HCFs stimulated with 0.1 μg/ml or 2.0 μg/ml of LPS (**a-d**). **f** displays representative images of Western blot.

To further elucidate the effects of RB-PDT on LPS induced KC-HCFs, we examined the expression of key molecules, involved in the NF-κB and MAPK signaling pathways. The transcriptional and phosphorylation levels of IKBKB, NF-κB, ERK-1, ERK-2, p38, and JNK are presented at **Figs 5 and 6**. RB-PDT significantly upregulated the transcriptional level of IKBKB and JNK in KC-HCFs without LPS stimulation (**Fig 5A and 5E**, p≤0.01). However, in the presence of LPS, RB-PDT did not demonstrate an effect on the transcriptional and phosphorylation levels of these NF-κB and MAPK pathway related molecules.

## Discussion

PDT can promote the formation of covalent bonds between collagen molecules, thereby increasing the crosslinking level of the corneal stroma [18,29]. Simultaneously, the reactive oxygen species (ROS) generated during this process are crucial for eliminating and inhibiting pathogens in keratitis [11,30,31]. In the field of ophthalmology, riboflavin-UVA PDT is the most widely utilized method. This regimen has demonstrated efficacy in slowing the progression of keratoconus and preventing the need for further keratoplasty [11,32,33]. Additionally, several studies have reported on its anti-microbial properties, indicating its potential in treating infectious keratitis [16,34,35]. However, there are several issues that need to be considered. Firstly, riboflavin-UVA PDT is effective in the corneal stroma approximately until 400 μm depth, suggesting its unsuitability for patients with thin corneas [33,36,37]. Secondly, there have been various case reports indicating the side effects of riboflavin-UVA PDT for corneal crosslinking, including corneal edema, haze and keratitis [38–40].

In contrast, RB-PDT exhibits similar capabilities in enhancing corneal rigidity but possesses novel characteristics. Firstly, the effect of RB-PDT extends up to 200 μm in the corneal stroma, thereby avoiding damages of the corneal endothelium, particularly in thinner corneas [17,36]. Secondly, the wavelength used in RB-PDT typically ranges between 530–570 nm, avoiding potential damage or side effects associated with UVA light, such as stimulating the reactivation of latent herpes simplex virus (HSV) [40,41]. It is also important to note that, based on previous research, the separate use of different fluences of green light or different concentrations of RB solution alone does not impact viability and proliferation of HCFs and KC-HCFs [42]. In our present study we could demonstrate that LPS concentration alone does not significantly change cell viability at concentrations of 0.1 to 2.0 μg/mL, but at higher concentrations (5.0 μg/mL), it may affect cell viability in combination with RB-PDT. Additionally, RB-PDT demonstrates superior anti-microbial effects compared to riboflavin-UVA PDT, particularly against fungal and acanthamoeba infections [21,43–45].

In this study, we employed KC-HCFs, stimulated with different concentrations of LPS to mimic infectious keratitis, followed by the application of RB-PDT treatment. Our findings indicate that RB-PDT could upregulate pro-inflammatory cytokine and chemokine expression during inflammation.

Using 0.1 μg/ml LPS stimulation and RB-PDT, the expression level of the pro-inflammatory cytokines IL-1β and IL-6 was upregulated, which was accompanied by an increased transcription of the IL-8 chemokine. In corneal inflammation, the IL-1β, IL-6, and IL-8 levels showed a significant correlation with the degree of inflammation [46]. IL-1β is renowned for its pro-inflammatory effects and can promote the programmed death of keratocytes, helping to localize infections [47]. Similarly, an increase in IL-6 levels amplifies the inflammatory response. Nevertheless, the elevated IL-6 levels in the short-term are protective for the cornea in combating bacterial invasion [48–50]. For example, in *Pseudomonas aeruginosa* infections, the administration of exogenous IL-6 significantly reduces bacterial counts, and IL-6^-/-^ mice exhibit more severe disease manifestations compared to their wild-type counterparts [51]. IL-8, as a chemokine, plays a pivotal role in keratitis by mediating the rapid recruitment of a significant number of neutrophils, mast cells, and T cells to the site of infection [52]. IL-1β, IL-6, and IL-8 can exhibit synergistic effects in the development of keratitis, eliminating pathogens and further mediating adaptive immune response [53]. In the present study, the expression of IL-1β, IL-6, and IL-8 increased 24 hours following RB-PDT treatment, indicating that RB-PDT can further amplify the inflammatory effect induced by LPS stimulation on KC-HCFs. It is noteworthy that the ELISA results for IL-1β and IL-8 did not demonstrate a significant increase in line with their transcription levels, which may be attributed to the potential temporal variations in proinflammatory cytokine levels [46,53].

Among the other measured cytokines and chemokines, only ICAM-1 exhibited a transcription increase after RB-PDT. However, if LPS was applied, RB-PDT did not amplify ICAM-1 expression. Therefore, we suppose that the primary pro-inflammatory cytokines affected by RB-PDT in LPS induced KC-HCFs inflammation are IL-1β, IL-6, and IL-8.

In our previous research and other literatures, the signal transduction of the Toll-like receptor 4 (TLR4) signaling pathway has been described in detail [24,48,54,55]. In the context of Gram-negative keratitis, LPS is recognized by TLR4 and triggers a cascade of downstream signaling pathways, the NF-κB and MAPK pathways, leading to the activation of the innate immune response.

Despite the observed increase in cytokines after RB-PDT, the NF-κB and MAPK pathways remained unaffected, as evidenced by the unaltered phosphorylation levels of key proteins such as NF-κB, p38, and JNK (**Fig 6**). In previous studies, we employed a similar approach using RB-PDT to treat LPS-induced healthy human fibroblasts (HCFs), at comparable concentrations. Our findings revealed a significant increase in IL-6 and IL-8 levels following RB-PDT treatment, accompanied by elevated transcription and phosphorylation of NF-κB, JNK, and p38 at the same time point [24]. This suggested a pronounced stimulatory effect of RB-PDT on TLR4-related signaling pathways in HCFs. However, such an effect was not observed in KC-HCFs. We speculate that this discrepancy may be attributed to the suspected presence of chronic inflammation in corneas of KC-HCFs patients.

Chronic inflammatory states in the cornea can contribute to the development of various diseases, including dry eye disease [56,57] and allergic conjunctivitis [57]. Under chronic inflammatory conditions, KC-HCFs may exhibit an altered immune response to external stimuli (e.g., RB-PDT), whereas HCFs maintain a normal response capability. Consequently, KC-HCFs did not demonstrate the same activation of TLR4-related pathway following RB-PDT as observed in HCFs. Furthermore, various reports have documented the occurrence of bacterial [58,59], fungal [60], viral [61,62], and sterile [63] keratitis in patients following corneal crosslinking, regardless of whether they adhered to the traditional "Dresden protocol" [40,58] or underwent accelerated protocol [34]. Researchers have attributed these observations to the removal of the corneal epithelium prior to crosslinking, UVA irradiation, or the use of bandage contact lenses [41,58]. Our study findings indicate a distinct immune response of KC-HCFs to RB-PDT compared to HCFs, potentially providing another perspective. The altered activation of TLR4-related pathway in KC-HCFs may underlie the pathogenesis of potential secondary keratitis following crosslinking. This may be attributed to the fact that the normal activation of TLR4 enables the prompt elimination of pathogens as well as facilitates the proliferation and migration of stromal cells, which does not happen in KC-HCFs [48,64]. A previous study has demonstrated that the inhibition of TLR4 leads to delayed corneal healing [64].

Following keratitis, the occurrence of haze may significantly impair the patients’ vision which may further necessitate corneal transplantation. In this study, we observed that in LPS-induced KC-HCFs inflammation, the transcription of collagen I and collagen V decreased after RB-PDT. Collagen I and collagen V are the primary ECM molecules in the corneal stroma, and their excessive accumulation can increase corneal opacity and may cause corneal scarring [65,66]. Furthermore, LOX, the enzyme that catalyzes the physiological crosslinking of collagen in the corneal stroma, remained unaffected after RB-PDT [67]. However, it is also important to note that in keratoconus, the expression of collagens I, and V may be decreased [68]. Furthermore, LOX expression and activity are reduced in keratoconus corneas [67,69]. These alterations in the corneal stroma associated with keratoconus may partially explain why collagen I, collagen V, and LOX protein bands were not detected in our samples using Western blot analysis.

In our findings, RB-PDT treatment of LPS-stimulated KC-HCFs increased the transcription of TGF-β1. TGF-β1 promotes the differentiation of fibroblasts into myofibroblasts, which facilitates collagen synthesis and corneal healing [70,71]. However, this transcriptional upregulation of TGF-β by RB-PDT was not observed at the protein expression level. Transcription and translation are integral components of the central dogma, yet discrepancies between transcription and translation levels are frequently observed. This may be attributed to the fact that the regulation of gene expression in organisms occurs not only at the transcriptional level but also involves multiple processes, such as post-transcriptional regulation, and post-translational regulation [72–74]. These regulatory mechanisms collectively determine the ultimate protein expression levels. Furthermore, the degradation rates, stability and turnover of mRNA and proteins can potentially influence their expression levels [75,76]. For instance, mRNA degradation may lead to decreased abundance, while protein degradation or modification may affect its stability and activity. Additionally, since we collected cells and cell supernatants at the same time point, temporal differences between transcription and translation may exist [77]. Similarly, the discrepancies observed between the transcription and translation results for IL-1β and IL-8 may also be related to these factors.

Furthermore, In the context of TGF-β, it is typically secreted as part of a complex with latent TGF-β-binding proteins (LDBPs). This complex anchors TGF-β within the ECM, thereby limiting its availability in the supernatant. As a result, when cellular supernatants are collected for analysis, the TGF-β produced by fibroblasts may not be fully released into the supernatant, making its detection by ELISA challenging [78]. Similarly, IL-1β and IL-8 are subject to tight regulatory mechanisms upon release to control inflammatory responses. For instance, IL-1β can bind to soluble IL-1 receptors (sIL-1R), inhibiting its activity and promoting its degradation [79]. IL-8, in contrast, is endocytosed by target cells after binding to CXCR1/CXCR2 receptors, followed by degradation [80]. These regulatory mechanisms help to explain the partial discordance observed between qPCR and ELISA. The availability of an animal model for keratoconus, combined with a longer experimental duration, could offer deeper insights into collagen synthesis.

There are some important limitations of the present study. The study only includes short-term observations, specifically at the 24-hour mark, which was selected based on prior experiments examining cell viability and proliferation [42]. Previously, using 0.14 J/cm^2^ fluence and 24 hours observation, RB-PDT still did not significantly affect KC-HCF viability [42]. Nevertheless, in the present study, RB-PDT with 0.14 J/cm^2^ fluence alone significantly decreased KC-HCF viability. This may be attributed to the use of primary cells, where factors such as the age of individual donors and the severity of keratoconus could influence the behavior of the isolated KC-HCFs in culture. Furthermore, in clinical settings, patients with severe infectious keratitis typically receive broad-spectrum antibiotics and, at times, low-dose corticosteroid eyedrops to prevent complications such as rapid corneal stroma dissolution and perforation. The potential interactions between RB-PDT and these treatments, particularly antibiotics and corticosteroids, were not explored in this study.

In addition, while in vivo experiments would provide a more comprehensive understanding of RB-PDT’s impact, keratoconus is currently only observed in humans, and there are limited animal models available for research. This limitation restricts the ability to investigate the therapy’s effects in a living system, but future studies may focus on developing suitable models to address this gap.

In conclusion, in LPS-induced KC-HCFs inflammation, the application of RB-PDT enhances IL-6 synthesis in the short term, which may provide advantages in corneal infectious disease. In LPS-induced inflammation of KC-HCFs treated with RB-PDT, despite the increased expression of pro-inflammatory cytokines, the activation of the TLR4 signaling pathways is lacking. RB-PDT may have no adverse effects on corneal scar formation of keratoconus corneas in the short term.

## Supporting information

S1 File (PDF)

S1 Raw images (PDF)
