# Supplementary material for: The cellular response of lipopolysaccharide-induced inflammation in keratoconus human corneal fibroblasts to RB-PDT: Insights into cytokines, chemokines and related signaling pathways
Source: PLoS One. 2025 Jan 27;20(1):e0318132. doi: 10.1371/journal.pone.0318132 (PMC11771863; doi:10.1371/journal.pone.0318132)
Supplement: S1 File — (PDF) [file pone.0318132.s001.pdf]

XTT	Only LPS					LPS + PDT				
0 μg/ml	1.07	1.14	1.6	1.21	1.03	0.68	0.85	1.06	0.88	0.9
0.1 μg/ml	1.1	1.08	1.33	0.98	0.92	0.76	0.69	0.82	0.75	1.08
0.25 μg/ml	1.07	1.08	1.52	1.1	1.07	0.69	0.86	1.14	0.9	0.72
0.5 μg/ml	1.21	1.17	1.36	1.06	0.97	0.85	0.72	1.18	0.72	0.77
1.0 μg/ml	1.03	1.05	1.5	1.13	0.91	0.71	0.74	1.17	0.88	0.9
2.0 μg/ml	1.15	1.15	1.4	1.07	0.89	0.78	0.73	0.83	0.77	1.04
5.0 μg/ml	1.01	1.07	1.49	1.11	1.05	0.7	0.79	0.97	0.8	0.85
10 μg/ml	1.11	1.18	1.53	1.15	1.1	0.85	0.87	1.03	0.79	0.99

PCR

NFκB	Only LPS					LPS + PDT				
0 μg/ml	1.32	0.62	0.89	1.13	1.04	1.71	1.38	1.35	1.27	1.36
0.1 μg/ml	1.76	1.8	0.81	0.97	1.35	1.09	1.73	1.08	0.79	1.42
2.0 μg/ml	1.5	1.92	0.87	1.18	1.19	2.03	1.82	0.97	1.21	1.43

MAPK 1	Only LPS					LPS + PDT				
0 μg/ml	0.75	0.93	0.93	1.39	1	0.93	1.25	1.05	1.32	1.15
0.1 μg/ml	0.73	1	0.92	1.29	1.21	0.78	0.93	0.84	1.44	1.13
2.0 μg/ml	0.79	1.09	0.93	0.92	0.91	0.84	1.1	0.85	1.23	1.32

MAPK 3	Only LPS					LPS + PDT				
0 μg/ml	0.9	0.83	1.21	1.26	0.8	1.42	0.44	0.3	0.54	0.8
0.1 μg/ml	0.84	1.31	0.82	1.02	0.7	0.82	0.83	0.54	0.3	0.76

2.0 µg/ml	1.06	1.55	1.07	1.17	0.85	1.02	1.16	0.62	0.26	0.99

MAPK 8	Only LPS					LPS + PDT				
0 µg/ml	0.68	0.89	1.18	1.03	1.22	0.91	1.93	2.09	1.48	2.18
0.1 µg/ml	0.77	1.55	1.38	1.34	1.62	1.09	1.32	1.35	2.12	1.01
2.0 µg/ml	0.89	1.61	1.25	0.97	1.32	0.84	1.46	1.43	1.2	1.45

MAPK 14	Only LPS					LPS + PDT				
0 µg/ml	1.05	1.01	0.87	1.12	0.96	1.12	1.04	1.73	1.14	1.02
0.1 µg/ml	0.82	1.21	1.45	0.97	0.92	1.03	1.31	1.5	1.08	0.9
2.0 µg/ml	1.07	1.22	1.38	0.99	0.83	1.06	1.15	1.33	1.19	1.1

IFNA2	Only LPS					LPS + PDT				

0 μg/ml	1.99	1.05	0.39	0.57		0.22	2.21	0.94	0.3	1.38
0.1 μg/ml	*4.07**	0.32	1.4	1.69		0.9	0.51	1.42	0.13	0.51
2.0 μg/ml	1.5	1.19	0.65	0.42	0.59	0	2.35	0.82	0.29	1.26

IFNB1			Only LPS					LPS + PDT		
0 μg/ml	1.44	1.04	0.72	0.9	0.91	0.46	0.59	0.98	1.01	0.63
0.1 μg/ml	1.29	0.68	0.86	0.78	1.35	1.07	1.22	0.66	0.58	1.17
2.0 μg/ml	0.79	0.78	0.45	0.48	1.09	0.59	1.03	0.43	0.76	1.11

COL1A1			Only LPS					LPS + PDT		
0 μg/ml	0.7	0.18	1.67	1.13	1.32	1.21	1.79	1.39	1.44	0.19
0.1 μg/ml	1.23	2.14	0.62	0.77	1.79	0.23	0.58	0.96	0.12	0.63
2.0 μg/ml	1.51	3.17	1.08	2.35	1.31	0.74	0.45	0.72	0.79	1.12

COL5A1			Only LPS					LPS + PDT		
0 µg/ml	1.15	0.99	0.94	0.9	1.02	1.08	1.2	0.78	0.75	1.27
0.1 µg/ml	1.11	1.13	0.73	0.75	0.92	0.68	0.87	0.53	0.57	0.7
2.0 µg/ml	1.13	1.42	0.59	1.07	1.25	0.8	0.94	0.58	0.56	0.73

LOX			Only LPS					LPS + PDT		
0 µg/ml	1.14	1.04	0.7	1.4	0.71	0.82	0.89	0.72	1	0.65
0.1 µg/ml	1.09	0.83	0.63	1.28	0.6	0.79	0.74	0.6	0.97	0.49
2.0 µg/ml	1.1	0.75	0.73	1.12	0.68	0.98	0.78	0.62	0.93	0.59

TGFbeta			Only LPS					LPS + PDT		
0 µg/ml	1	0.85	1.09	0.91	1.14	1.09	1.32	1.55	1.09	1.29
0.1 µg/ml	0.73	0.76	0.93	0.75	0.95	0.97	1.18	1.01	1.23	1.27
2.0 µg/ml	0.86	0.8	0.77	0.68	0.77	0.88	1.08	0.83	1.01	1.13

Western blot

p-ERK / ERK	Only LPS					LPS + PDT				
0 μg/ml	1	1	1	1	1	0.65	1.69	1.77	1.3	1.34
0.1 μg/ml	0.84	1.6	1.67	0.92	1.5	0.86	1.75	1.63	1.57	1.51
2.0 μg/ml	0.54	1.66	1.26	1.1	1.4	0.67	1.4	1.42	1.41	1.51

p-JNK /JNK	Only LPS					LPS + PDT				
0 μg/ml	1	1	1	1	1	0.43	1.78	1.54	0.74	0.79
0.1 μg/ml	0.74	1.77	1.1	1.25	1.39	0.55	1.76	1.52	1.01	1.1
2.0 μg/ml	0.3	1.64	1.21	0.78	1.24	0.44	1.77	1.35	0.83	0.92

p-p38 / p38	Only LPS					LPS + PDT				
0 μg/ml	1	1	1	1	1	0.61	1.41	1.43	1.1	0.84
0.1 μg/ml	0.71	1.37	1.22	1.07	1.09	0.63	1.5	1.15	1.3	1.22
2.0 μg/ml	0.55	1.48	1.03	1.11	1.04	0.52	1.3	1.13	1.26	1.18

p-NFκB / NF κB	Only LPS					LPS + PDT				
0 μg/ml	1	1	1	1	1	0.29	1.48	1.69	0.89	1.11
0.1 μg/ml	0.54	1.29	1.55	0.65	1.37	0.48	1.71	1.71	1.23	1.36

2.0 μg/ml	0.31	1.62	1.66	1.14	1.33	0.49	1.5	1.72	1.3	1.34

ICAM1	Only LPS					LPS + PDT				
0 μg/ml	1	1	1	1	1	4.63	1.43	1.76	1.69	1.98
0.1 μg/ml	12.34	2.3	11.53	2.75	3.98	8.83	1.87	7.55	2.15	3.72
2.0 μg/ml	11.26	4.2	12.09	3.65	3.95	12.46	3.46	12.21	3.46	5.67

ELISA

IL-1β			Only LPS					LPS + PDT		
0 μg/ml	7.96	4.94	3.22	3.99	4.22	7.28	4.91	3.67	4.82	5.1
0.1 μg/ml	8.67	7.19	4.44	5.19	5.52	10.43	12.69	4.18	6.06	6.43
2.0 μg/ml	11.01	7.86	4.17	5.13	4.69	8.96	7.63	4.15	5.16	4.96

IL-6			Only LPS					LPS + PDT		
0 μg/ml	19.7	18.02	3.72	6.37	5.6	38.48	31.82	16.67	22.3	10.85
0.1 μg/ml	29.61	32.28	21.17	17.76	14.27	67.16	77.7	34.03	38.43	38.5
2.0 μg/ml	44.75	43.05	30.7	27.45	23.58	70.61	54.79	40.27	36.42	34.85

IL-8			Only LPS				LPS + PDT			
0 μg/ml	73	56.84	35.41	28.92		76.84	68.33	40.83	34.54	
0.1 μg/ml	59.41	31	38.1	30.39		79.55	33.08	26.29	29.96	

2.0 μg/ml	43.48	16.41	18.63	40.73		52.68	15.89	18.58	40.06	

CCL4	Only LPS					LPS + PDT				
0 μg/ml	7.69	5.82	4.4	2.16	1.85	7.47	4.7	3.23	5.11	1.81
0.1 μg/ml	7.26	5.59	3.91	3.55	1.94	9.67	6.2	3.09	1.65	2.96
2.0 μg/ml	8.51	5.74	2.63	2.11	3.1	6.53	4.12	3.05		1.87

TGF-β	Only LPS					LPS + PDT				
0 μg/ml	106.79	67.77	61.49	59.83	70.14	68.5	55.15	43.91	50.6	58.6
0.1 μg/ml	75.26	53.17	58.3	59.48	58.13	79.86	56.88	37.04	46.47	60.58
2.0 μg/ml	70.72	59.42	48.28	56.62	54.7	60.53	40.17	34.88	43.75	40.35
