# Supplementary material for: The cellular response of lipopolysaccharide-induced inflammation in keratoconus human corneal fibroblasts to RB-PDT: Insights into cytokines, chemokines and related signaling pathways
Source: PLoS One. 2025 Jan 27;20(1):e0318132. doi: 10.1371/journal.pone.0318132 (PMC11771863; doi:10.1371/journal.pone.0318132)
Supplement: S1 Raw images — (PDF) [file pone.0318132.s002.pdf]

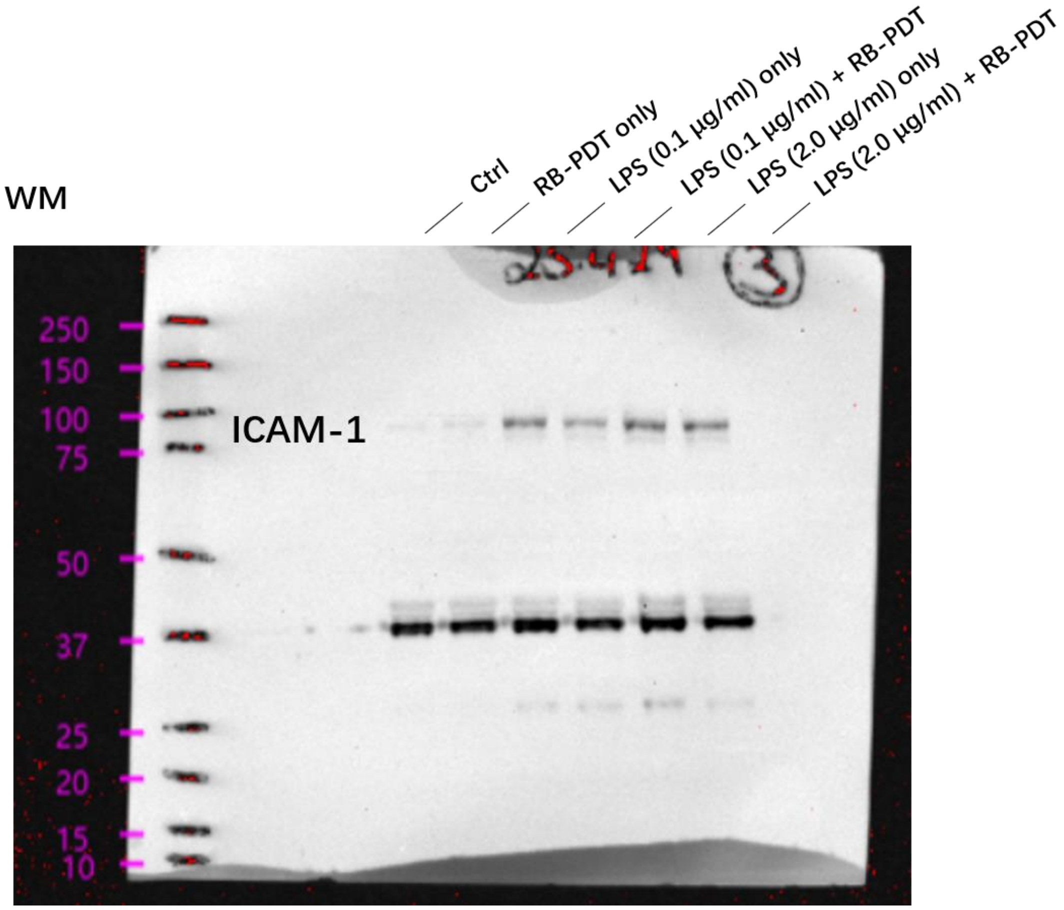
WM
Ctrl
RB-PDT only
LPS (0.1 μg/ml) only
LPS (0.1 μg/ml) + RB-PDT
LPS (2.0 μg/ml) only
LPS (2.0 μg/ml) + RB-PDT
250
150
100
75
50
37
25
20
15
10
ICAM-1

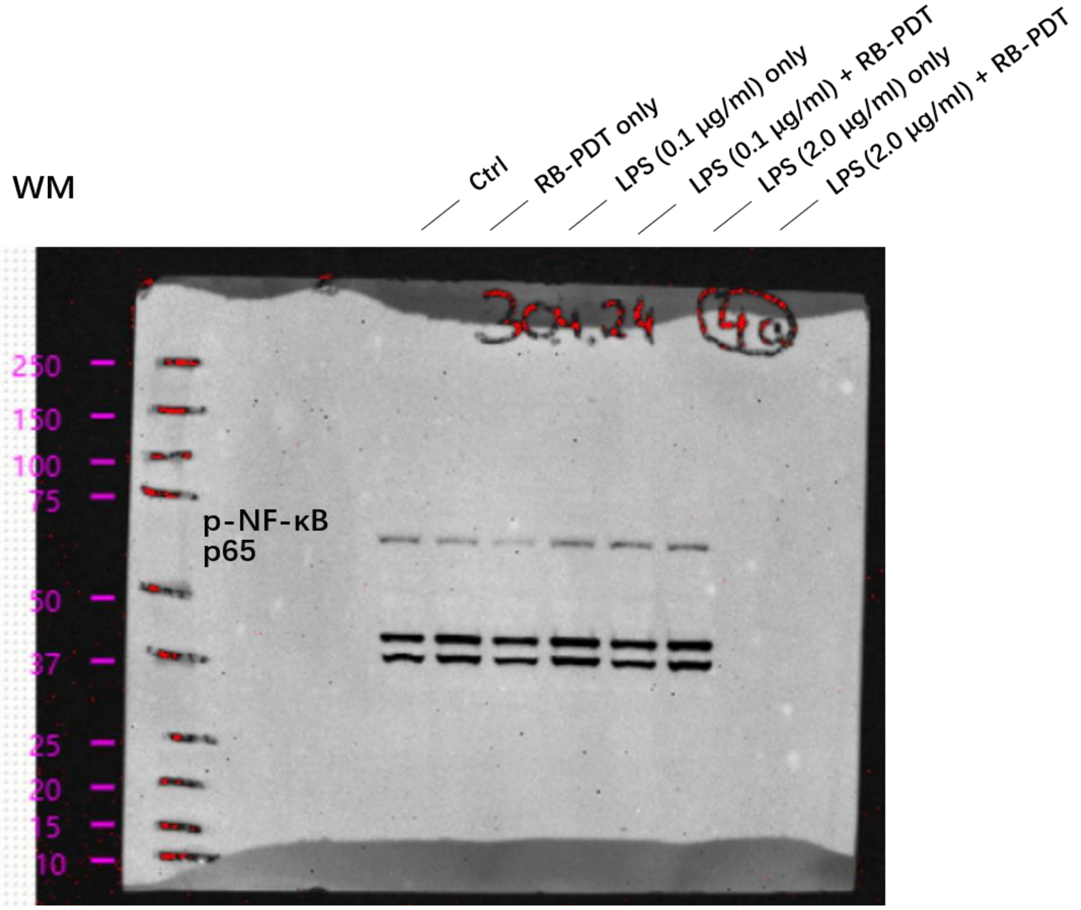
WM
Ctrl
RB-PDT only
LPS (0.1 µg/ml) only
LPS (0.1 µg/ml) + RB-PDT
LPS (2.0 µg/ml) only
LPS (2.0 µg/ml) + RB-PDT
250
150
100
75
50
37
25
20
15
10
p-NF-κB
p65

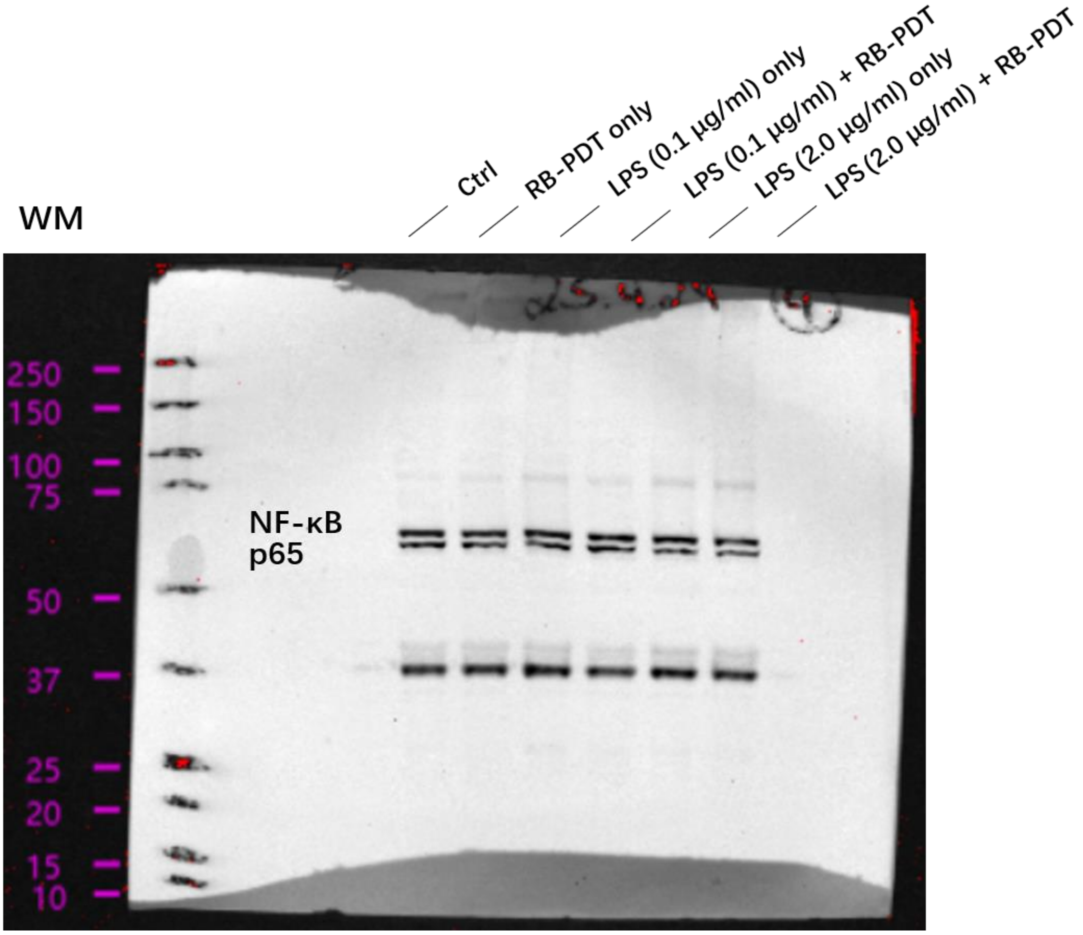
WM
Ctrl
RB-PDT only
LPS (0.1 µg/ml) only
LPS (0.1 µg/ml) + RB-PDT
LPS (2.0 µg/ml) only
LPS (2.0 µg/ml) + RB-PDT
250
150
100
75
50
37
25
20
15
10
NF-κB
p65

WM

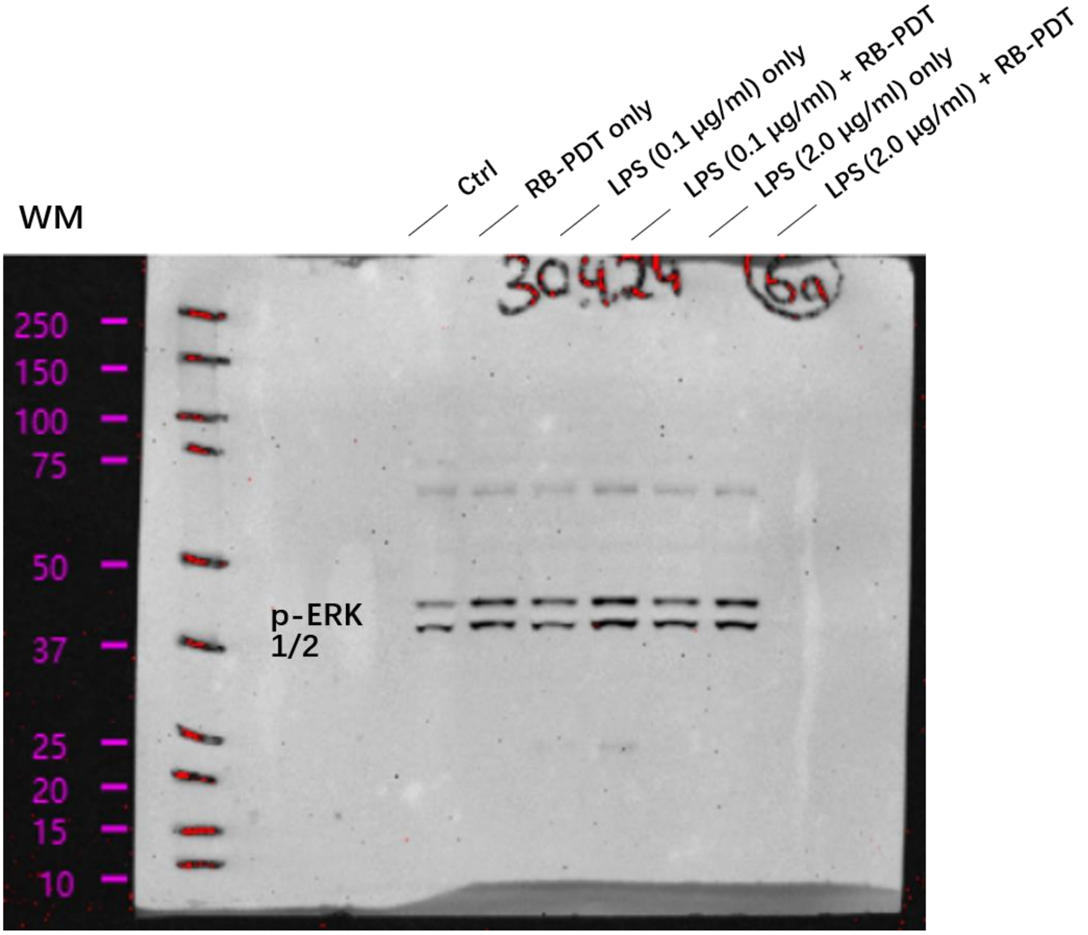

Ctrl
RB-PDT only
LPS (0.1 µg/ml) only
LPS (0.1 µg/ml) + RB-PDT
LPS (2.0 µg/ml) only
LPS (2.0 µg/ml) + RB-PDT
250
150
100
75
50
37
25
20
15
10
p-ERK 1/2
304.24
6a

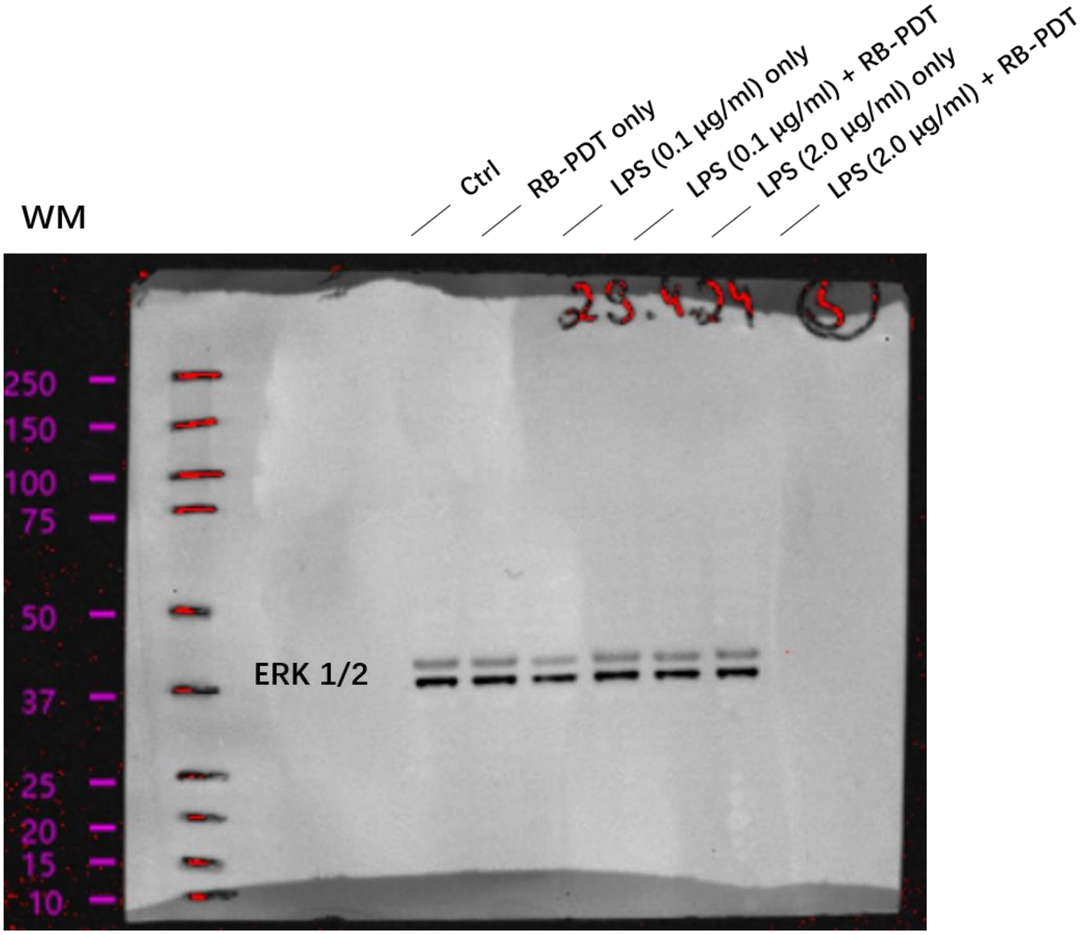

WM
Ctrl
RB-PDT only
LPS (0.1 µg/ml) only
LPS (0.1 µg/ml) + RB-PDT
LPS (2.0 µg/ml) only
LPS (2.0 µg/ml) + RB-PDT
250
150
100
75
50
37
25
20
15
10
ERK 1/2

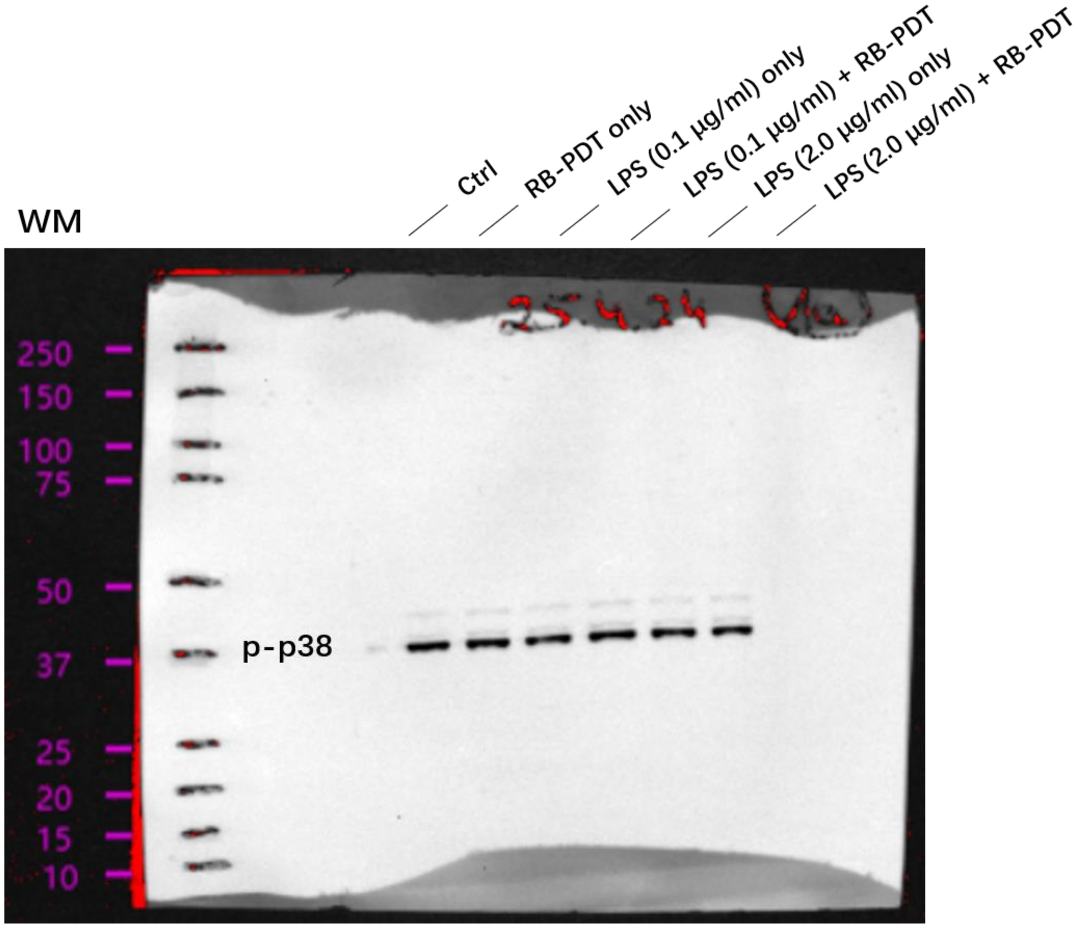
WM
Ctrl
RB-PDT only
LPS (0.1 µg/ml) only
LPS (0.1 µg/ml) + RB-PDT
LPS (2.0 µg/ml) only
LPS (2.0 µg/ml) + RB-PDT
250
150
100
75
50
37
25
20
15
10
p-p38

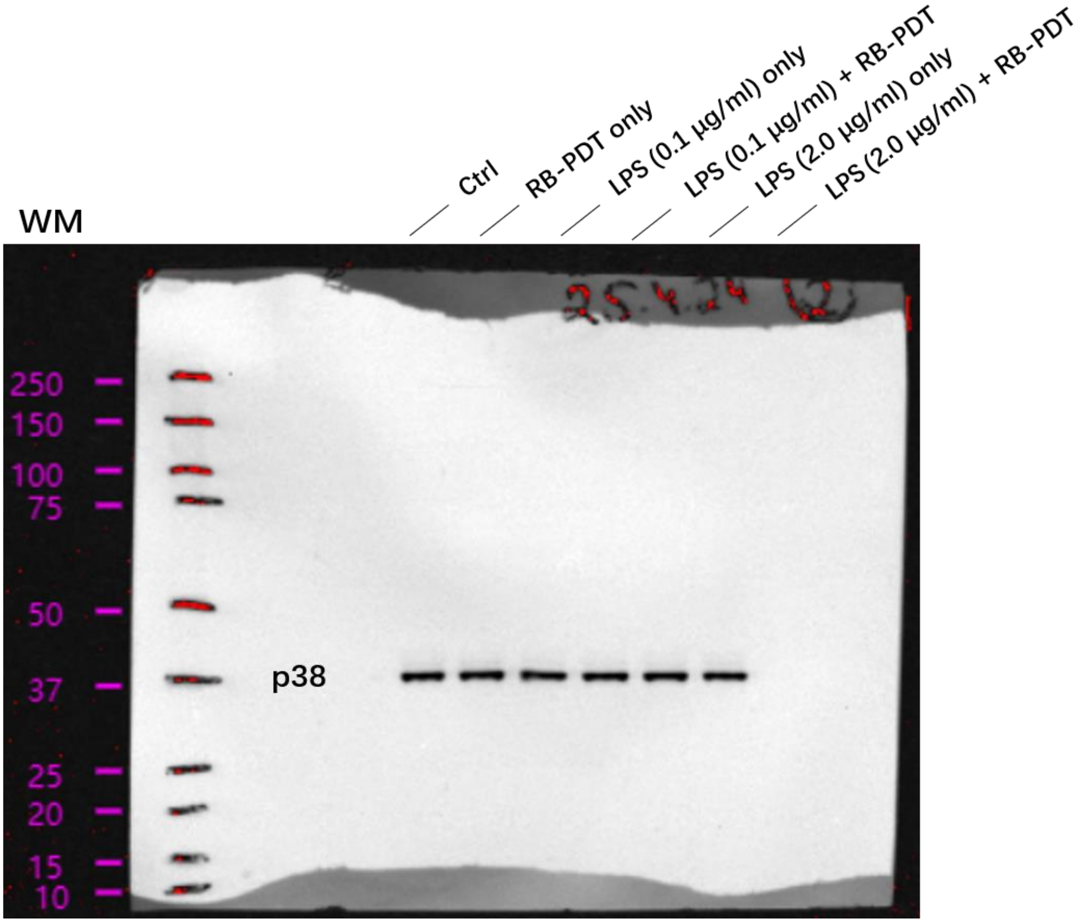

WM
Ctrl
RB-PDT only
LPS (0.1 µg/ml) only
LPS (0.1 µg/ml) + RB-PDT
LPS (2.0 µg/ml) only
LPS (2.0 µg/ml) + RB-PDT
250
150
100
75
50
37
25
20
15
10
p38

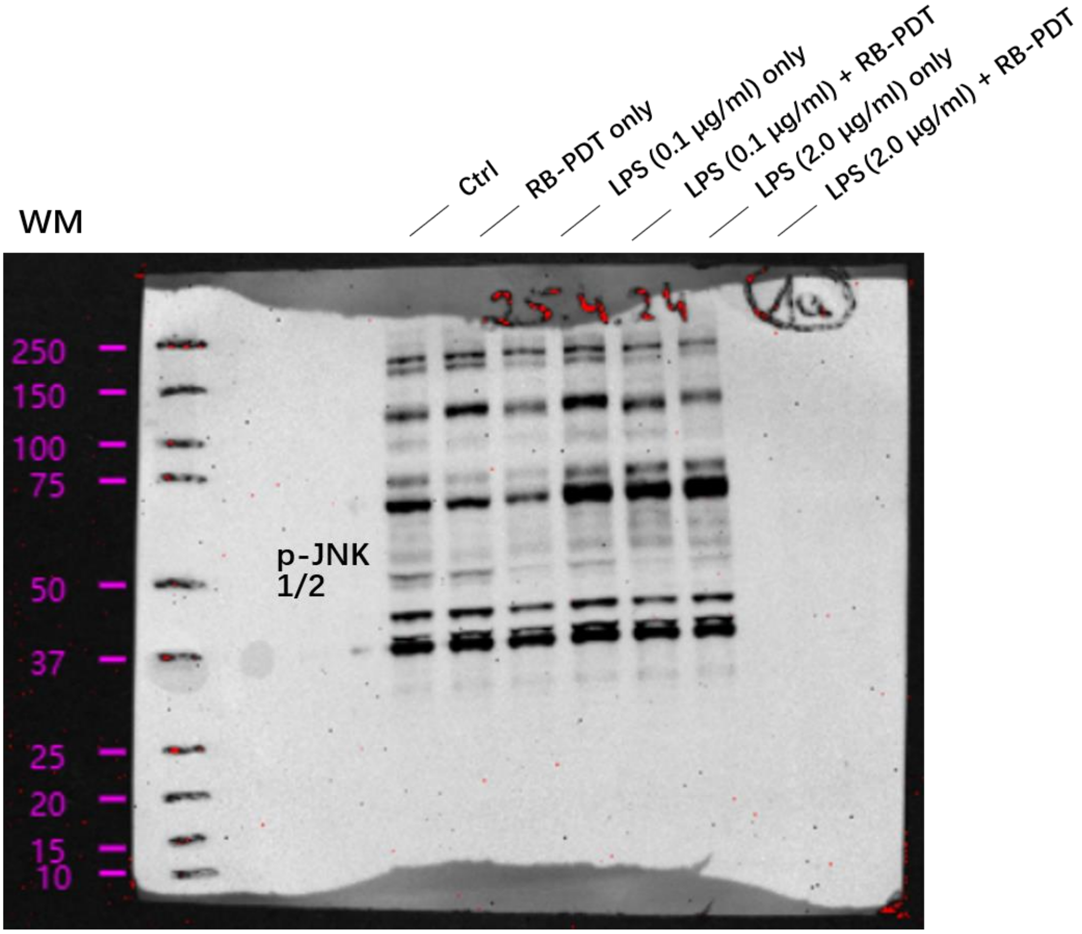
WM
Ctrl
RB-PDT only
LPS (0.1 µg/ml) only
LPS (0.1 µg/ml) + RB-PDT
LPS (2.0 µg/ml) only
LPS (2.0 µg/ml) + RB-PDT
250
150
100
75
50
37
25
20
15
10
p-JNK 1/2
25.4.24

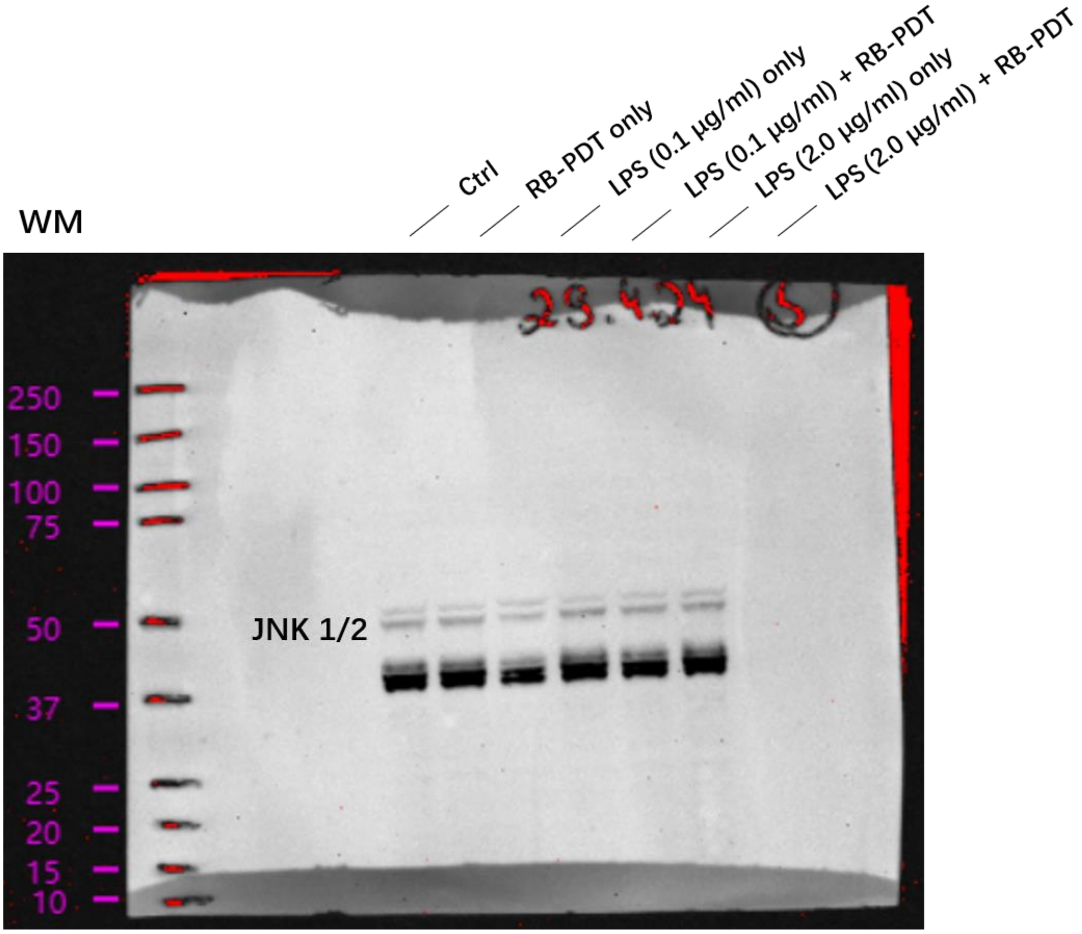

WM
Ctrl
RB-PDT only
LPS (0.1 μg/ml) only
LPS (0.1 μg/ml) + RB-PDT
LPS (2.0 μg/ml) only
LPS (2.0 μg/ml) + RB-PDT
250
150
100
75
50
37
25
20
15
10
JNK 1/2

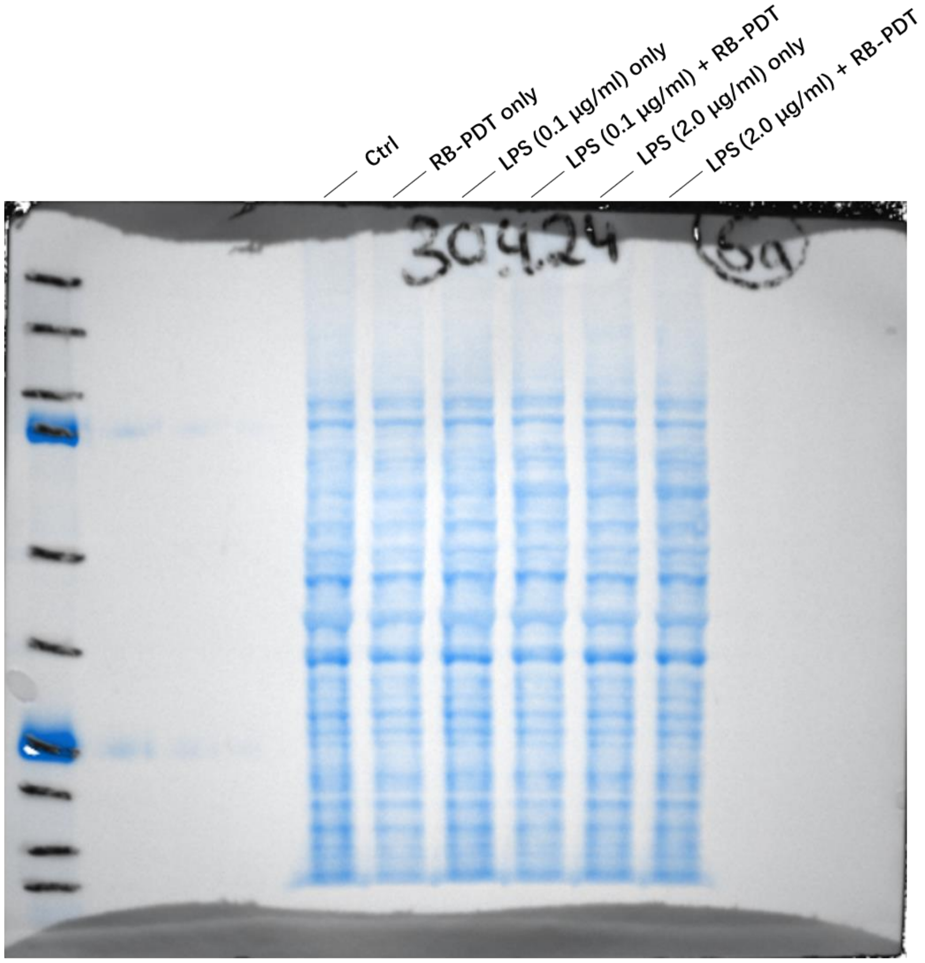

Groups	Ctrl	RB-PDT only	LPS (0.1 μg/ml)	LPS (0.1 μg/ml) + RB-PDT	LPS only (2.0 μg/ml)	LPS (2.0 μg/ml) + RB-PDT
TPN factor	1	0.890	1.117	0.955	0.988	0.984
